# Modulation of cherry tomato performances in response to molybdenum biofortification and arbuscular mycorrhizal fungi in a soilless system

**DOI:** 10.1016/j.heliyon.2024.e33498

**Published:** 2024-06-22

**Authors:** Lorena Vultaggio, Enrica Allevato, Leo Sabatino, Georgia Ntatsi, Youssef Rouphael, Livio Torta, Salvatore La Bella, Beppe Benedetto Consentino

**Affiliations:** a Department of Agricultural, Food, and Forestry Sciences (SAAF), University of Palermo, 90128 Palermo, Italy; b Department of Environmental and Prevention Sciences (DiSAP), University of Ferrara, 44121 Ferrara, Italy; c Department of Crop Science, Laboratory of Vegetable Production, Agricultural University of Athens, 11855 Athens, Greece; d Department of Agricultural Sciences, University of Naples Federico II, Portici, Italy

**Keywords:** *Solanum lycopersicum* L., *Glomus intraradices*, Mo-enrichment, Trace element distress, Hazard quotient, Functional food

## Abstract

Molybdenum (Mo) is a crucial microelement for both, humans and plants. The use of agronomic biofortification techniques can be an alternative method to enhance Mo content in vegetables. Concomitantly, arbuscular mycorrhizal fungi (AMF) application is a valuable strategy to enhance plant performances and overcome plant abiotic distresses such as microelement overdose. The aim of this research was to estimate the direct and/or indirect effects of Mo supply at four doses [0.0, 0.5 (standard dose), 2.0 or 4.0 μmol L^−1^], alone or combined with AMF inoculation, on plant performances. In particular, plant height and first flower truss emission, productive features (total yield, marketable yield and average marketable fruit weight) and fruit qualitative characteristics (fruit dry matter, soluble solids content, titratable acidity, ascorbic acid, lycopene, polyphenol, nitrogen, copper, iron and molybdenum) of an established cherry tomato genotype cultivated in soilless conditions were investigated. Moreover, proline and malondialdehyde concentrations, as well as Mo hazard quotient (HQ) in response to experimental treatments were determined. A split-plot randomized experimental block design with Mo dosages as plots and +AMF or -AMF as sub-plots was adopted. Data revealed that AMF inoculation enhanced marketable yield (+50.0 %), as well as some qualitative traits, such as fruit soluble solids content (SSC) (+9.9 %), ascorbic acid (+7.3 %), polyphenols (+2.3 %), and lycopene (+2.5 %). Molybdenum application significantly increased SSC, polyphenols, fruit Mo concentration (+29.0 % and +100.0 % in plants biofortified with 2.0 and 4.0 μmol Mo L^−1^ compared to those fertigated with the standard dose, respectively) and proline, whereas it decreased N (−25.0 % and −41.6 % in plants biofortified with 2.0 and 4.0 μmol Mo L^−1^ compared to those fertigated with the standard dose, respectively). Interestingly, the application of AMF mitigated the detrimental effect of high Mo dosages (2.0 or 4.0 μmol L^−1^). A pronounced advance in terms of plant height 45 DAT, fruit lycopene concentration and fruit Fe, Cu and Mo concentrations was observed when AMF treatment and Mo dosages (2.0 or 4.0 μmol Mo L^−1^) were combined. Plants inoculated or not with AMF showed an improvement in the hazard quotient (HQ) in reaction to Mo application. However, the HQ - for a consumption of 200 g day^−1^ of biofortified cherry tomato - remained within the safety level for human consumption. This study suggests that Mo-implementation (at 2.0 or 4.0 μmol L^−1^) combined with AMF inoculation could represent a viable cultivation protocol to enhance yield, produce premium quality tomato fruits and, concomitantly, improve Mo dose in human diet. In the light of our findings, further studies on the interaction between AMF and microelements in other vegetable crops are recommended.

## Highlights

• Mo-biofortification combined with AMF enhanced tomato marketable yield by 50 % compared to the control.
• Arbuscular mycorrhizal fungi inoculation increased Mo-biofortification efficiency by 12.8 %.
• Mo supply and arbuscular mycorrhizal fungi improved tomato nutritive and functional traits.

## 1 Introduction

Tomato (*Solanum lycopersicum* L.) is a plant belonging to the nightshade (*Solanaceae*) family, which also encloses other important vegetables such as pepper, eggplant and potato. Tomato is a crop widely cultivated and its fruits are used for fresh consumption and for processed foods production with relevant economic and nutritional aspects and contributing - significantly - to food security and global economy [1]. Tomato had its origin in the south American Andes, and subsequently, was brought to Europe, Asia and Africa by Spanish conquerors from the 16th century onwards [2]. Currently - on a worldwide basis - tomato production is mainly localized in Asia, America, Turkey and Italy [3]. According to the most recent data, the total world production in 2021 was around 190 million tonnes, with China in the first place producing over 60 million tonnes, which representing the 31.6 % of the global production [3]. Italy is a leading tomato producer in Europe with a production of 6 million tonnes in 2021. In this scenario, Apulia region leads with more than 2 million tonnes, followed by Sicily (632,038 t) [4].

Micronutrient shortage, also known as mineral malnutrition or hidden hunger, is a relevant problem affecting over 30 % of the world's population [5]. Large evidence indicates that a low consumption of vegetables and fruits, an unbalanced and unvaried diet along with overdosing of sub-standard cereals and processed foods, contribute to human nutritional deficits in many parts of the world [6,7]. Inadequate nutritional habits can lead to the onset of deficiencies of essential micronutrients like iron, vitamin A and iodine [[7], [8], [9], [10]], which in turn might have critical effects on human health, such as inefficient immune system, poor cognitive development, and high risk of diseases [11,12]. Therefore, although in the last year there has been large concern on many forms of human malnutrition (protein and calories deficiencies), micronutrient deficiency has now become a main challenge.

Plant biofortification is a promising strategy to increase crop nutritional value through different methods (plant breeding, genetic engineering and agronomic practices) [13]. Plant biofortification can be achieved by two different strategies: genetic or agronomic. Genetic biofortification strategy uses classical genetic improvement or modern biotechnology to increase micronutrients concentration in plant edible parts, whereas agronomic biofortification consists in the supply of microelements, via foliar spray or fertigation [5]. Production of microelement enriched vegetables via agronomic biofortification programs represents not only an area of readily applicable research, but also a valuable, sustainable and profitable strategy to overcome micronutrient shortage in humans, particularly in the vulnerable population [[14], [15], [16]].

Molybdenum (Mo) is a trace element vital for plants, animals and microbes [17], which could be enclosed as a core trace element in agronomic biofortification programs [15,18,19]. Molybdate (MoO_4_^2−^) is the most common Mo chemical form employed in agronomic biofortification programs [15,19]. This Mo form is active in plants as a pterin complex called molybdopterin, which produces the Mo cofactor (Moco) [20]. Moco is imperative for the cycles of nitrogen, carbon, sulphur, and hormone biosynthesis in plants [[20], [21], [22], [23]]. The fundamental enzymes connected to Mo are nitrate reductase and aldehyde oxidase. The first one is implied in the reduction of nitrate in nitrite [24], whereas, aldehyde oxidase partakes in the biosynthesis of hormones, like abscisic acid and indole-3-acetic acid, which affect plant growth and development [[25], [26], [27]]. Molybdenum is also involved in the chlorophyll biosynthesis pathway, which in turn is strictly related to plant photosynthesis [17]. Indeed, Mo deficiency can lead to reduced growth and yield and yellowing of the leaves [28]. Usually, an appropriate quantity of Mo is present in the soil, but it is only adequate for plant growth. Consequently, farmers rarely supply this microelement in standard fertilisation programmes for open field cultivations. As a result, Mo concentration in plants could be quite low and not sufficient to assure beneficial effects on human health [[29], [30], [31]], especially when plants are cultivated in soilless systems. Therefore, Mo biofortification is an effective and alternative method to increase Mo concentration in plant edible organs to obtain a positive influence on human health [5,[32], [33], [34]].

Currently, the use of environmentally friendly agronomic practices to safeguard crop production and quality under top, sub-optimal or critical conditions is of paramount importance [35] and, in this respect, the use of biostimulants could be a valuable aid [36]. Among microbial biostimulants, arbuscular mycorrhizal fungi (AMF) are often employed in agriculture. They are telluric fungi that establish a symbiotic association with many plant species [37], including vegetables. These microorganisms enhance the uptake of several minerals (phosphorus, nitrogen, sulphur, potassium, calcium, copper and zinc) from the rhizosphere and transfer them to the host plant via a dense system of hyphae which acts as an auxiliary absorption system. In response, host plants provide to the AMF with sugars that they would otherwise be unable to synthesise as chemoheterotrophic organisms [38,39]. The AMF are also recognized as plant stress alleviators [[39], [40], [41]]. For abiotic stresses, the AMF adaptation mechanisms are mostly correlated to enhanced mineral nutrition, ion uptake regulation, gene functioning and osmolytes, phytohormones and antioxidants biosynthesis [42]. According to Diagne et al. [42]. AMF inoculation has relevant role in pathogen resistance comprising competition for colonization spots and enhancement of the plant's defence system. Moreover, Kumar et al. [43] observed that AMF have a considerable role in the mitigation of heavy metal distress through a physical control mechanism. Sbrana et al. [44] and Weisany [45] also claimed that AMF symbiosis can modify plant primary and secondary metabolism, increasing the biosynthesis of phytochemicals.

**Fig. 1:**
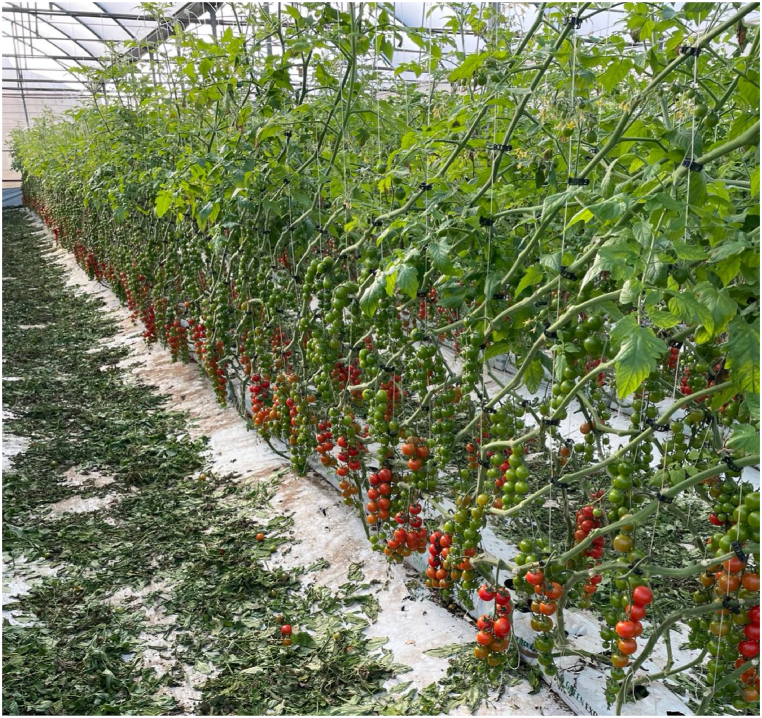
Morphological aspects of the plants under study.

The effect of Mo-biofortification and AMF application on vegetable crops has been investigated [5,[46], [47], [48], [49]], however, there are no reports concerning their synergistic effect. Since AMF can alleviate mineral toxicity stress [39] and, considering that Mo can be toxic to plants at high concentrations [19,50,51], we suppose that the combined AMF and Mo supply might affect plant growth, yield and overall plant performances. However, the mechanism of Mo toxicity in tomato soilless cultivation as influenced by AMF inoculation remains unknown. For the above premise, the aim of the present research was to assess the effect of Mo supply at four different concentrations on AMF inoculated or non-inoculated plants of cherry tomato grown in a soilless system. Specifically, plant growth, yield, nutritional and functional traits, as well as proline, malondialdehyde concentration and Mo hazard quotient were appraised (Fig. 1).

## 2 Materials and methods

### 2.1 Experimental site and plant material

The research was performed for two consecutive years (2019 and 2020) in an experimental farm of the Department of Agricultural, Food and Forestry Sciences (SAAF) of the University of Palermo. The experimental site was located at Marsala, province of Trapani, (longitude 12°26′E, latitude 37°47′N, altitude 37 m). The ‘Tyty’ F_1_ hybrid (Syngenta Seed, Basel, Switzerland) of cherry tomato (*Solanum lycopersicum* var. Cerasiforme) was tested in a high-tech greenhouse covered with polyvinyl chloride and provided with a fan-cooling, an over-head air heating and a high-pressure fogging systems.

### 2.2 Tomato cultivation technique

On February 18, 2019 and 2020, tomato plug plants at a stage of 5 true leaves were transplanted in coconut fibre/perlite (70/30, v/v) based substrate mix bags [Agripan perlite (Perlite Italiana, Milan, Italy)], obtaining 3.3 plants m^−2^. All tomato plants were fertigated with the same nutrient solution (Table 1.).

**Table 1:** Nutrient solution characteristics for soilless tomato cultivation.

Nutrient solution composition													
Macronutrients (mM)							Micronutrients (μM)					EC (mS cm^−1^)	pH
NH_4_^+^	K^+^	Ca^2+^	Mg^2+^	NO_3_^−^	SO_4_^2-^	H_2_PO_4_	Fe	Mn	Zn	B	Cu		
1.2	9.5	5.4	2.4	16.0	4.4	1.5	15.0	10.0	5.0	30.0	0.75	3.60	5.6

The nutrient solution pH was measured every day and properly regulated by adding an adequate amount of HNO_3_ to obtain a pH value ranging from 5.7 to 5.9. Plant nutrition was accomplished via a drip irrigation system with in-line drippers (2.0 L h^−1^). Water volumes were evaluated based on knowledge of the preceding day's solar radiation [52,53], imposing a leaching fraction of 35 %. The cultivation was conducted as an open soilless system. Greenhouse's climatic conditions were regulated by an informatic system. The air temperature inside the high-tech greenhouse was imposed to 16 ± 1 °C overnight and 24 ± 1 °C throughout the day, while the relative humidity was maintained in a range of 60–70 % during the whole growing period, in order to prevent low fruit set due to pollination issues.

### 2.3 Arbuscular mycorrhizal inoculation and molybdenum application

Twenty-four hours prior to transplanting into the perlite bags, the root system was soaked in the arbuscular mycorrhizal fungi (AMF) inoculum solution for 10 min with the strain CMCCROC7 (Bioplanet, Cesena, Italy) of *Rhizophagus irregularis* (fomerly *Glomus intraradices*), employing a dose of 400 spores per plant. With regard to biofortification, molybdenum (Mo) was supplied at four different levels [0.0, 0.5 (standard dose), 2.0, and 4.0 μmol L^−1^], from beginning to the end of the experiment. To achieve different Mo levels in the nutrient solution, appropriate quantities of ammonium molybdate tetrahydrate (Merck KGaA, Darmstadt, Germany) were added.

### 2.4 Experimental design and statistics

The four Mo levels (0.0 0.5, 2.0, and 4.0 μmol L^−1^) were combined with AMF inoculation [-AMF (not-inoculated) or + AMF (inoculated)] in a two-factor split-plot experimental design (Mo dosages as plots and +AMF or -AMF as sub-plots) which generated eight treatments, each replicated 4 times (8 plants per replicate, resulting in a total of 256 plants). Statistical analysis was performed by SPSS software, version 28.0 (StatSoft, Inc., Chicago, USA) using the general linear model (GLM). A preliminary three-way Analysis Of Variance (ANOVA), considering year, Mo dosage and AMF inoculation as main factors was performed to evaluate the effect of the year. Since ANOVA did not underline a statistically effect of the year, the Mo-biofortification and AMF inoculation effects were evaluated via a two-way ANOVA, setting Mo dose and AMF inoculation as the main factors. For values expressed as percentage, the arcsin transformation was applied before ANOVA analysis [Ø = arcsin (p/100)^1/2^]. Mean separation was accomplished via Tukey HSD test at *p* ≤ 0.05. A principal component analysis (PCA) was also carried out to assess any primary association among Mo doses and AMF inoculation on all recorded traits. Only principal components (PCs) with eigenvalues higher than 1.0 were selected. Moreover, a heatmap of the correlation coefficients for all parameter was realized via the online program package ClustVis (https://biit.cs.ut.ee/clustvis/).

### 2.5 Mycorrhizal colonization, plant vigour and yield components

To assay mycorrhizal colonization, the approach described by Giambalvo et al. [54] was adopted. Mycorrhizal inoculation was determined at fruiting stage, and it was shown as a percentage of infection.

Tomato plant height at 45 days after transplanting (DAT) and the emission of the first flower truss, expressed as DAT, were recorded. After the harvest, total and marketable yield (expressed as kg plant^−1^) were measured, moreover average fruit weight (g fruit^−1^) was also calculated.

### 2.6 Fruit dry matter, soluble solids content, titratable acidity, ascorbic acid, polyphenol and lycopene concentration

All determinations on fruit quality were assessed on 5 samples randomly chosen from each replicate from the 2nd and 3rd harvest. Only non-damaged fruits were chosen. For the evaluation of fruit dry matter, the samples after being cut were placed inside an oven set at 105 °C until a constant weight. The dry matter content was reported as a percentage. As reported by Sabatino et al. [55] a digital refractometer (MTD-045 nD, Taipei, Taiwan) was used to determine the soluble solids content (SSC) of the tomato juice; the value was then expressed as °Brix. For the titratable acidity (TA) analysis, the method of Han et al. [56] was followed, thus aliquots of 10 g of cherry tomatoes were placed in 50 mL of distilled water and titrated with 0.1 N NaOH until a final pH of 8.1 was reached. The TA was expressed as percentage of citric acid. The SSC/TA ratio was also calculated.

The ascorbic acid content in cherry tomato plants was assessed following the method reported by Sabatino et al. [55] via a Reflectometer RQflex10 Reflectoquant® (Sigma-Aldrich, Saint Louis, MO, USA). Briefly, 1 g of tomato juice was dissolved in 10 mL of distilled water, then with the aid of Reflectoquant Ascorbic Acid test strip (Merck, Darmstadt, Germany) values were revealed and reported as mg ascorbic acid per 100 g of fresh weight (fw). The polyphenol concentration in cherry tomato fruit was established using Folin-Ciocalteu colorimetric method [57]. The standard or sample extract (100 μL; triplicate) was mixed with 0.4 mL Folin-Ciocalteu reagent. After 3 min reaction 0.8 mL of 10 % Na_2_CO_3_ was added. After allowing the tubes to stand for 30 min at room temperature, absorption was measured at 765 nm using a spectrophotometer (CELL, model CE 1020, Cambridge, UK). Gallic acid was used as a standard for calibration. Data were showed as gallic acid equivalent [GAE mg 100 g^−1^ dry weight (dw)]. Fruit lycopene concentration was determined following the method described by Sadler et al. [58]. Briefly, 5 g of homogenised sample was extracted adding 50 mL of a mixture of hexane/acetone/ethanol (2:1:1, v/v/v) for half hour. Total lycopene fruit content was obtained by measuring the absorbance at 472 nm of the lycopene hexane fraction. Pure lycopene (Sigma, St. Louis, MO) was used for the preparation of calibration curves. The values were expressed in mg 100 g^−1^ dry weight.

### 2.7 Nitrogen, iron, copper and molybdenum concentrations of tomato fruits

Determinations on fruit minerals were conducted on 5 samples randomly chosen from each replicate from the 2nd and 3rd harvest. The Kjeldahl method was followed to assess nitrogen (N) concentration in tomato fruits. Digestion was carried out on Labtec DT 220 with the concomitant use of Scrubber Labtec SR 210, while distillation was performed with Tecator Kjeltec 8200 (FOSS A/S, Hillerød, Denmark). Then, the total fruit N content was determined by manual titration for each distilled sample, measuring the ml of HCl solution (0.05 N) required to turn the colour of the solution from green to pink. Values were reported as g 100 g^−1^ dry weight. Atomic absorption spectroscopy (SavantAA, ERRECI, Milan, Italy) was adopted to determinate content of microelements such as iron (Fe) and copper (Cu) in cherry tomato fruits, following wet mineralization as described by Morand and Gullo [59]. Thus, atomic absorption spectroscopy (SavantAA, 200 ERRECI, Milan, Italy) was adopted. Fruit Mo concentration was assessed as reported by Sabatino et al. [19] by ICP-MS instrument (Plasma Quant MS Elite, Jena, Germany), roughing pump, re-circulator, data acquisition and analysis software, equipped with a low liquid uptake nebulizer, a free-running radio frequency (RF) plasma generator, automated X, Y, Z torch positioning, and a four-stage vacuum system. A MARS6 (CEM, USA) high throughput closed microwave digestion workstation was adopted for dissolving metal and preparing reference solution. Fruit iron (Fe), copper (Cu) and Mo concentrations were expressed as mg kg^−1^ dry weight.

### 2.8 Proline and malondialdehyde content

According to Li et al. [60] method, the malondialdehyde (MDA) content (nmol g^−1^ fw) was evaluated. Briefly, approximately 0.5 g of leaf sample was taken and immersed in 1.5 mL of trichloroacetic acid (5 %). Centrifugation at 5000*g* for 10 min was performed and then the volume of supernatant was made up to 10 mL. Two mL were extracted and homogenised with 2 mL of thiobarbituric acid (0.67 %). The mixture was immersed in hot water (100 °C) for 30 min and centrifugated again for 10 min at 5000 g. Finally, the absorbances of the aqueous phase were measured at 450, 532 and 600 nm. Proline content (μg g^−1^ fresh weight) in plant, was established according to the colorimetric method of Toscano et al. [61]. Briefly, proline was extracted from 1 g of samples mixed with 5 mL of aqueous sulphosalicylic acid (3 %). The mixture was then centrifugated for 15 min at 14,000 g, then 2 mL of this solution was homogenised with 2 mL of acetic acid and acid ninhydrin and incubated for 1 h at 100 °C. The reaction was completed in an ice bath and isolated with toluene. The extract was mixed for 20 s and the absorbance was measured with a spectrophotometer at 525 nm. Toluene was utilized as a blank while l-proline was used as standard.

### 2.9 Hazard quotient for biofortified tomato fruits

Following the Protocol of the United States Environmental Protection Agency (EPA), the Hazard quotient (HQ) of the biofortified tomato fruits was also determined using the following formula:

HQ = EDI/RfD

Where EDI is the daily intake of the Mo, expressed as μg day^−1^, while RfD is the level of acceptable Mo upper intake for humans, expressed as μg Mo day^−1^, set at 600 μg day^−1^. The HQ was calculated considering an adult person of 70 kg and a 200 g of daily portion of cherry tomato.

## 3 Results

As the statistical analysis did not show a significant effect of the year on all recorded parameters, data of the first growing cycle (2019) were presented. Data of the second experimental year (2020) were reported as supplementary materials (Tables S1–S5).

### 3.1 Inferences of AMF inoculation and molybdenum supply on fungal colonization rate, plant growth, first truss emission and yield features

**Fig. 2:**
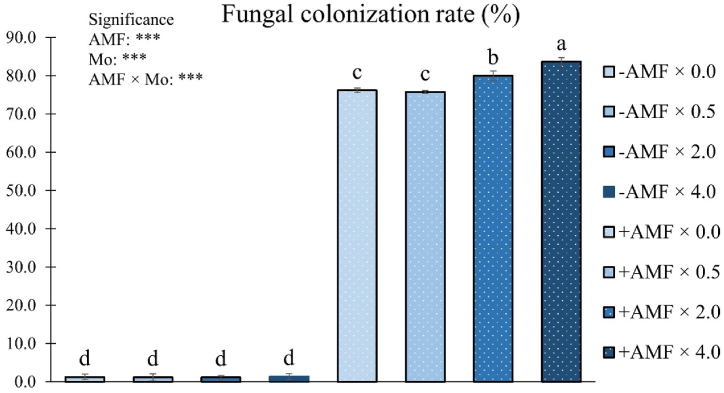
Fungal colonization rate percentage as influenced by arbuscular mycorrhizal fungi inoculation (AMF) and Mo biofortification (0.0, 0.5, 2.0 and 4.0 μmol L^−1^). Values with different letters are diverse according to Tukey HSD (*p* ≤ 0.05). ***: significant at *p* ≤ 0.001. Bars indicate mean ± SD. Mo: molybdenum; -AMF: non-inoculated; +AMF: inoculated with arbuscular mycorrhizal fungi.

ANOVA evidenced a significant interactive effect of AMF and Mo on fungal colonization rate (Fig. 2).

The percentage of colonization peaked at 83.7 % in +AMF plants fertigated with 4.0 μmol Mo L^−1^, followed by the +AMF × 2.0 μmol Mo L^−1^ combination (80.0 %). The lowest percentages of fungal colonization rate (less than 2 %) were observed in -AMF plants (Fig. 2).

**Fig. 3:**
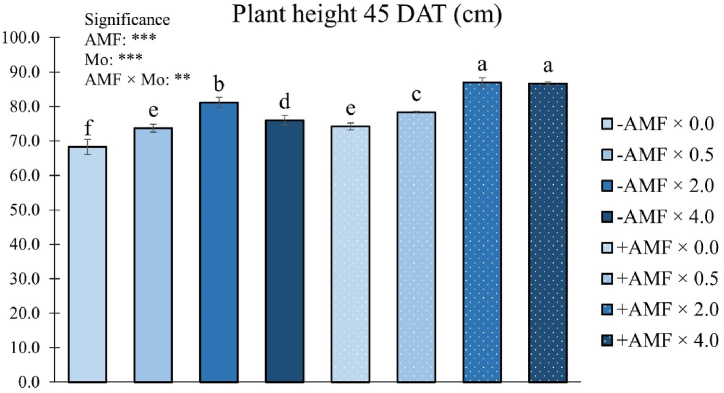
Plant height at 45 days after transplanting (DAT) as influenced by arbuscular mycorrhizal fungi inoculation (AMF) and Mo biofortification (0.0, 0.5, 2.0 and 4.0 μmol L^−1^). Values with different letters are diverse according to Tukey HSD (*p* ≤ 0.05). Bars indicate mean ± SD. ***: significant at *p* ≤ 0.001; **: significant at *p* ≤ 0.01. Mo: molybdenum; -AMF: non-inoculated; +AMF: inoculated with arbuscular mycorrhizal fungi.

There was a significant interaction AMF × Mo on plant height 45 DAT (Fig. 3).

Plants treated with AMF and exposed to Mo at 2.0 or 4.0 μmol L^−1^ revealed the highest height, followed by those not inoculated and biofortified with 2.0 μmol Mo L^−1^. The shortest plants were in the control plots (0.0 μmol Mo L^−1^ × -AMF combination), with an average height of 68.2 cm (Fig. 3).

Statistical analysis of first flower truss emission and yield traits did not underline an AMF × Mo interaction (Table 2).

**Table 2:** First truss emission, total yield, marketable yield and average marketable fruit weight as influenced by arbuscular mycorrhizal fungi inoculation (AMF), Mo biofortification and their interaction.

Treatments	First flower truss emission (DAT)	Total yield (kg plant^−1^)	Marketable yield (kg plant^−1^)	Average marketable fruit weight (g)
Mychorrhization				
-AMF	25.4 ± 1.4a	1.3 ± 0.1b	1.2 ± 0.1b	30.0 ± 1.4
+AMF	23.6 ± 1.1b	1.9 ± 0.2a	1.8 ± 0.2a	30.2 ± 0.7
*Mo doses (μmol L*^*−*^*^1^)*				
0.0	23.3 ± 1.0b	1.6 ± 0.4	1.5 ± 0.3	30.1 ± 1.4
0.5	23.4 ± 0.8b	1.6 ± 0.4	1.5 ± 0.3	30.3 ± 0.7
2.0	25.6 ± 1.2a	1.6 ± 0.3	1.5 ± 0.2	30.2 ± 0.9
4.0	25.8 ± 1.3a	1.8 ± 0.4	1.7 ± 0.4	29.9 ± 1.5
*Mychorrhization* × *Mo doses (μmol L*^*−*^*^1^)*				
-AMF × 0.0	24.1 ± 0.2	1.3 ± 0.2	1.2 ± 0.1	29.9 ± 2.1
-AMF × 0.5	24.1 ± 0.4	1.3 ± 0.1	1.1 ± 0.1	30.3 ± 0.8
-AMF × 2.0	26.7 ± 0.4	1.4 ± 0.1	1.3 ± 0.1	30.0 ± 1.2
-AMF × 4.0	26.9 ± 0.2	1.4 ± 0.2	1.3 ± 0.2	29.8 ± 2.2
+AMF × 0.0	22.5 ± 0.6	1.9 ± 0.3	1.7 ± 0.3	30.3 ± 0.8
+AMF × 0.5	22.8 ± 0.3	1.9 ± 0.1	1.8 ± 0.1	30.2 ± 0.9
+AMF × 2.0	24.5 ± 0.5	1.8 ± 0.1	1.7 ± 0.1	30.3 ± 0.8
+AMF × 4.0	24.7 ± 0.6	2.1 ± 0.1	2.0 ± 0.1	30.1 ± 0.8
*Significance*				
AMF	***	***	***	NS
Mo	***	NS	NS	NS
AMF × Mo	NS	NS	NS	NS

Data are presented as mean ± SD. Means with different letters are statistically dissimilar according to Tukey HSD test at *p* ≤ 0.05. ***: significant at *p* ≤ 0.001; NS: not statistically significant. Mo: molybdenum; -AMF: non-inoculated; +AMF: inoculated with arbuscular mycorrhizal fungi.

Regardless of Mo treatment, the application of AMF notably anticipated plant first flower truss emission (average 1.8 days) (Table 2). Non-biofortified plants and those supplied with 0.5 μmol Mo L^−1^ had the lowest first flower truss emission values, whereas Mo supplied at 2.0 or 4.0 μmol L^−1^ delayed first flower truss emission. AMF inoculation increased total and marketable yield by 46.1 % and 50.0 % compared with the control (-AMF), respectively; however, they were not affected by Mo supply (Table 2). Average marketable fruit weight was not significantly influenced either by AMF treatment, or by Mo-biofortification (Table 2).

### 3.2 Inferences of AMF inoculation and molybdenum supply on nutritional and functional traits

ANOVA for fruit dry matter, SSC, TA and SSC/TA did not highlight a significant interaction AMF × Mo (Table 3).

**Table 3:** Fruit dry matter percentage, soluble solids content (SSC), titratable acidity (TA) and SSC/TA ratio as influenced by arbuscular mycorrhizal fungi inoculation (AMF), Mo biofortification and their interaction.

Treatments	Fruit dry matter (%)	SSC (°Brix)	TA (% citric acid)	SSC/TA
*Mychorrhization*				
-AMF	6.6 ± 0.3	8.1 ± 0.3b	0.6 ± 0.03	14.5 ± 0.8b
+AMF	6.6 ± 0.3	8.9 ± 0.4a	0.6 ± 0.02	16.2 ± 0.7a
*Mo doses (μmol L*^*−*^*^1^)*				
0.0	6.4 ± 0.1c	8.0 ± 0.4c	0.5 ± 0.02	14.8 ± 1.2
0.5	6.4 ± 0.1c	9.0 ± 0.5a	0.6 ± 0.03	15.9 ± 0.7
2.0	6.7 ± 0.2b	8.5 ± 0.4b	0.6 ± 0.03	15.2 ± 1.4
4.0	7.0 ± 0.2a	8.4 ± 0.5b	0.5 ± 0.02	15.5 ± 1.1
*Mychorrhization* × *Mo doses (μmol L*^*−*^*^1^)*				
-AMF × 0.0	6.4 ± 0.1	7.7 ± 0.2	0.6 ± 0.03	13.9 ± 0.6
-AMF × 0.5	6.5 ± 0.1	8.5 ± 0.1	0.6 ± 0.03	15.4 ± 0.7
-AMF × 2.0	6.6 ± 0.1	8.1 ± 0.2	0.6 ± 0.03	14.1 ± 0.4
-AMF × 4.0	7.0 ± 0.2	8.0 ± 0.1	0.5 ± 0.03	14.7 ± 0.8
+AMF × 0.0	6.5 ± 0.1	8.4 ± 0.2	0.5 ± 0.02	15.8 ± 0.6
+AMF × 0.5	6.3 ± 0.2	9.5 ± 0.2	0.6 ± 0.02	16.4 ± 0.1
+AMF × 2.0	6.8 ± 0.2	8.8 ± 0.3	0.5 ± 0.03	16.3 ± 1.2
+AMF × 4.0	6.9 ± 0.2	8.9 ± 0.2	0.5 ± 0.02	16.2 ± 0.7
*Significance*				
AMF	NS	***	NS	***
Mo	***	***	NS	NS
AMF × Mo	NS	NS	NS	NS

Data are presented as mean ± SD. Means with different letters are statistically dissimilar according to Tukey HSD test at *p* ≤ 0.05. ***: significant at *p* ≤ 0.001; NS: not statistically significant. Mo: molybdenum; -AMF: non-inoculated; +AMF: inoculated with arbuscular mycorrhizal fungi.

Averaged over Mo doses, AMF treatment did not affect fruit dry matter percentage (Table 3). Inversely, regardless of the mycorrhization, plants subjected to the highest Mo dosage (4.0 μmol L^−1^) showed the highest fruit dry matter percentage (+9.3 % compared to non-biofortified plants), followed by those treated with 2.0 μmol Mo L^−1^. The lowest dry matter values were detected in fruits from plants fertigated with 0.0 or 0.5 μmol Mo L^−1^ (Table 3). Arbuscular mycorrhizal inoculation meaningfully increased fruit SSC by 0.8 °Brix (+9.8 %). Irrespective of the mycorrhization, fruits from plants supplied with 0.5 μmol Mo L^−1^ had the highest SSC (+12.5 % compared to non-biofortified plants), followed by those from plots fertigated with 2.0 or 4.0 μmol Mo L^−1^. Fruits from non-biofortified plants had the lowest SSC (Table 3). Fruit titratable acidity was not significantly affected by the main factors (Table 3). The ratio between SSC and TA was significantly enhanced by the AMF inoculation (+11.7 %), whereas Mo doses had no significant effect on SSC/TA (Table 3).

**Fig. 4:**
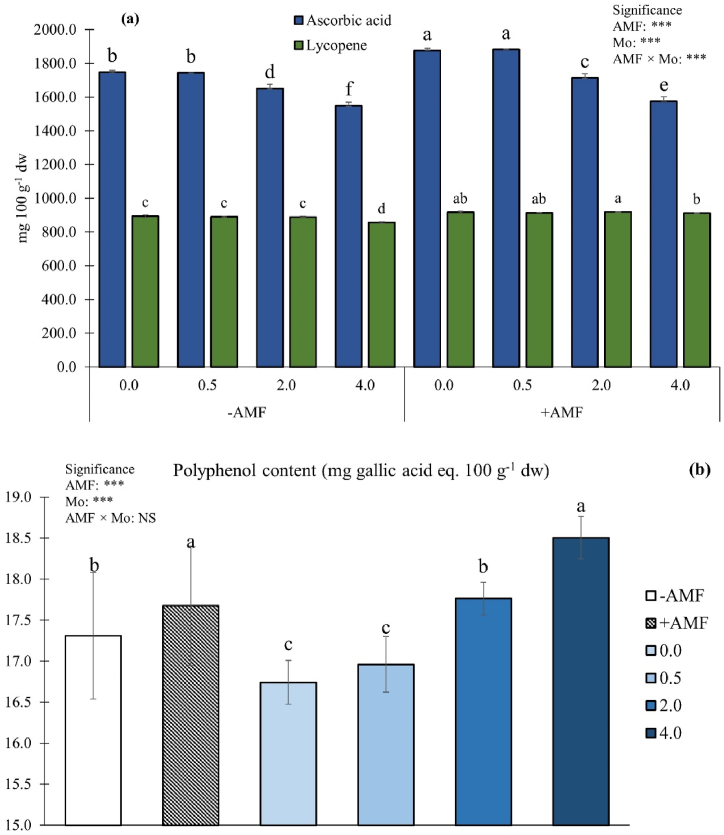
Ascorbic acid (a), lycopene (a) and polyphenols content (b) as influenced by arbuscular mycorrhizal fungi inoculation (AMF) and Mo biofortification (0.0, 0.5, 2.0 and 4.0 μmol L^−1^). Values with different letters are diverse according to Tukey HSD (*p* ≤ 0.05). Bars indicate mean ± SD. ***: significant at *p* ≤ 0.001; NS: not statistically significant. Mo: molybdenum; -AMF: non-inoculated; +AMF: inoculated with arbuscular mycorrhizal fungi.

For ascorbic acid, ANOVA revealed a statistically significant effect of the AMF × Mo interaction (Fig. 4a).

Overall, AMF inoculation increased fruit ascorbic acid, whereas the highest Mo dosages (2.0 and 4.0 μmol L^−1^) significantly decreased ascorbic acid concentration in both + AMF and -AMF plants (Fig. 4a). The highest ascorbic acid concentrations were detected in fruits from the +AMF × 0.0 or 0.5 μmol Mo L^−1^ combinations (7.3 % and 7.7 % compared to the control plants, respectively). Fruits from -AMF plants exposed to the highest Mo dose had the lowest ascorbic acid concentration (−11.4 % compared with the control plants) (Fig. 4a). Statistics for lycopene revealed that the main factors significantly interacted each other (Fig. 4a). Cherry tomato fruits from +AMF × 2.0 μmol Mo L^−1^ plots showed the highest values (+2.8 % compared with the control plants), whereas, when Mo supply was increased to 4.0 μmol L^−1^, lycopene concentration significantly decreased. However, inoculated plants biofortified with 0.0 or 0.5 μmol Mo L^−1^ did not significantly differ either from +AMF plants biofortified with 2.0 μmol Mo L^−1^, or from those treated with AMF and supplied with 4.0 μmol Mo L^−1^ (Fig. 4a). The lowest lycopene concentration was recorded in fruits from -AMF plants biofortified with the highest Mo dose (Fig. 4a). Polyphenol concentration was not influenced by the interaction AMF × Mo, however it was separately influenced by the two factors (Fig. 4b). Irrespective of the Mo application, AMF significantly enhanced polyphenols concentration by 2.2 % compared to the control. Fruits from plants exposed to the highest Mo dose had the highest polyphenols concentration, whereas the lowest values were found in plots supplied with 0.0 or 0.5 μmol Mo L^−1^ (Fig. 4b).

### 3.3 Inferences of AMF inoculation and molybdenum supply on fruit nitrogen, iron, copper and molybdenum concentrations

**Fig. 5:**
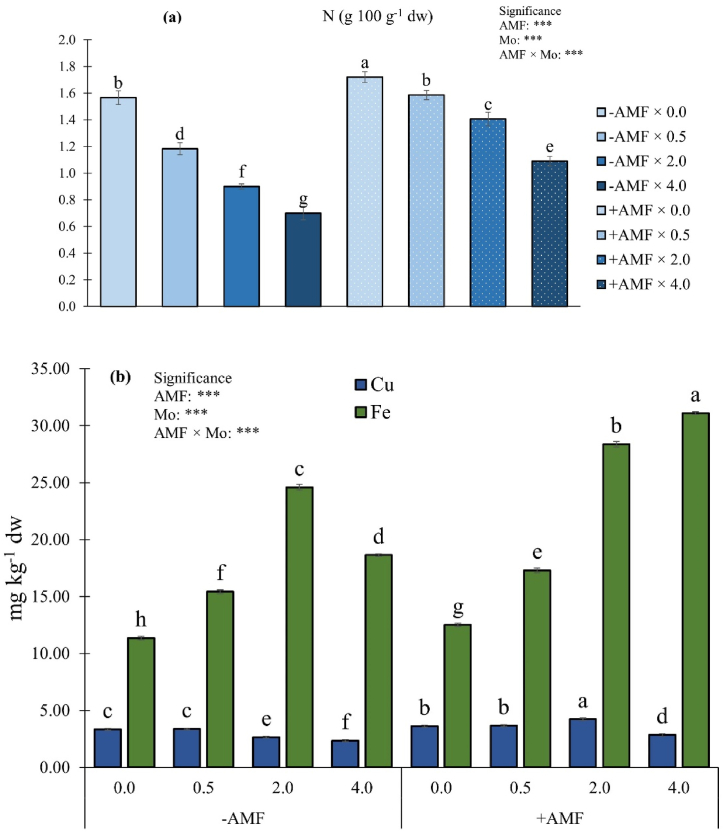
Nitrogen (a), iron and copper (b) fruit concentrations as influenced by arbuscular mycorrhizal fungi inoculation (AMF) and Mo biofortification (0.0, 0.5, 2.0 and 4.0 μmol L^−1^). Values with different letters are diverse according to Tukey HSD (*p* ≤ 0.05). Bars indicate mean ± SD. ***: significant at *p* ≤ 0.001. Mo: molybdenum; -AMF: non-inoculated; +AMF: inoculated with arbuscular mycorrhizal fungi.

Statistical analysis for fruit N, Fe and Cu concentrations pointed out a significant AMF × Mo interaction (Fig. 5a and b).

In both + AMF and -AMF plants, N fruit concentration decreased as Mo dose increased. Fruits from +AMF plants not biofortified with Mo had the highest N concentration (+6.3 % compared to the control) The lowest N concentrations were found in fruits from -AMF plants treated with the highest Mo dose (−56.2 % compared to the control) (Fig. 5a). Overall, as regard -AMF plants, the highest Fe concentration was observed in plants exposed to 2.0 μmol Mo L^−1^, whereas as regard + AMF plants the peak was detected at the highest Mo dosage (4.0 μmol Mo L^−1^) (Fig. 5b). The lowest fruit Fe concentration was recorded in the -AMF × 0.0 μmol Mo L^−1^ combination. Regarding Cu content, fruits from plots treated with 2.0 μmol Mo L^−1^ and inoculated with AMF had the highest concentration, while fruits from the -AMF × 4.0 μmol Mo L^−1^ combination had the lowest one.

**Fig. 6:**
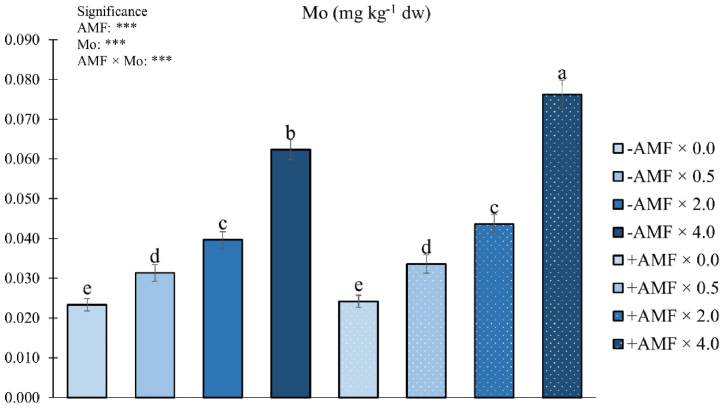
Fruit Mo concentration as influenced by arbuscular mycorrhizal fungi inoculation (AMF) and Mo biofortification (0.0, 0.5, 2.0 and 4.0 μmol L^−1^). Values with different letters are diverse according to Tukey HSD (*p* ≤ 0.05). Bars indicate mean ± SD. ***: significant at *p* ≤ 0.001. Bars indicate mean ± SE. Mo: molybdenum; -AMF: non-inoculated; +AMF: inoculated with arbuscular mycorrhizal fungi.

As reported in Fig. 6, the Mo concentration in fruits was significantly influenced by the AMF × Mo interaction.

Fruits from plants biostimulated with AMF and enriched with the highest Mo dosage showed the highest Mo concentration (+330 % compared to non-treated plants), followed by those from plots non-inoculated and biofortified with 4.0 μmol Mo L^−1^ (+270 % compared to non-treated plants). Not biofortified plants, either exposed or not to AMF inoculation, revealed the lowest Mo fruit concentration (Fig. 6).

**Fig. 7:**
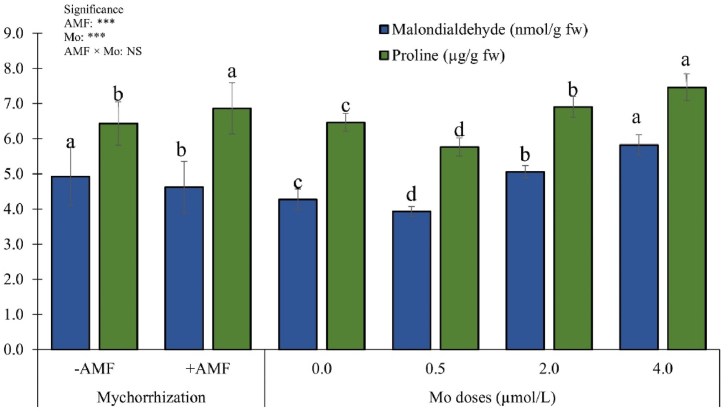
Malondialdehyde (MDA) and proline concentrations as influenced by arbuscular mycorrhizal fungi inoculation (AMF) and Mo biofortification (0.0, 0.5, 2.0 and 4.0 μmol L^−1^). Values with different letters are diverse according to Tukey HSD (*p* ≤ 0.05). Bars indicate mean ± SD. ***: significant at *p* ≤ 0.001; NS: not statistically significant. Mo: molybdenum; -AMF: non-inoculated; +AMF: inoculated with arbuscular mycorrhizal fungi.

Malondialdehyde and proline concentrations were not influenced by the interaction AMF × Mo, however they were individually affected by the two main factors (Fig. 7).

The malondialdehyde (MDA) concentration was meaningfully reduced by AMF inoculation (−6.1 %). Regardless of the AMF treatments, plants fertigated with the highest Mo dose revealed the highest MDA concentration, followed by those fertigated with 2.0 μmol Mo L^−1^. Plants from plots treated with 0.5 μmol Mo L^−1^ showed the lowest MDA concentration (Fig. 7). Plant proline concentration was significantly enhanced by AMF inoculation (+7.8 %). Regardless of the AMF application, plants exposed to 4.0 μmol Mo L^−1^ revealed the highest proline values. The lowest proline concentration was observed in plants fertigated with 0.5 μmol Mo L^−1^ (Fig. 7).

**Fig. 8:**
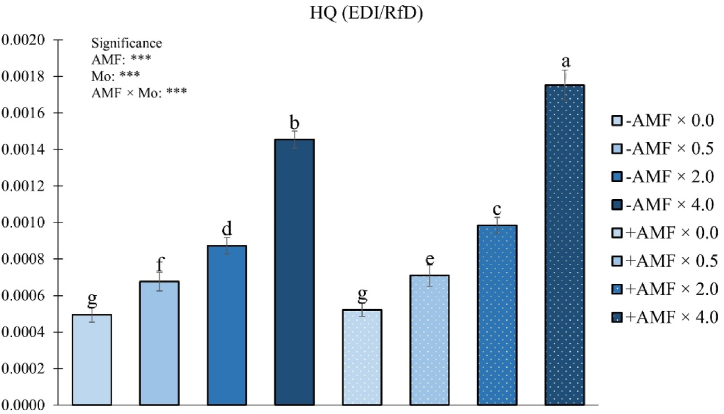
Mo hazard quotient (HQ) as influenced by arbuscular mycorrhizal fungi inoculation (AMF) and Mo biofortification (0.0, 0.5, 2.0 and 4.0 μmol L^−1^). Values with different letters are diverse according to Tukey HSD (*p* ≤ 0.05). Bars indicate mean ± SD. ***: significant at *p* ≤ 0.001. Mo: molybdenum; -AMF: non-inoculated; +AMF: inoculated with arbuscular mycorrhizal fungi.

Molybdenum hazard quotient (HQ) was meaningfully affected by the AMF × Mo interaction (Fig. 8).

All treatments revealed HQ values within the safety range (<1) (Fig. 8). Fruits from +AMF plants and biofortified with the highest Mo dose revealed the highest HQ values, followed by those treated with the same Mo dose but not inoculated with the biostimulant. The lowest HQ values were found in fruits from +AMF and -AMF plants fertigated with 0.0 μmol Mo L^−1^ (Fig. 8).

### 3.4 Principal component analysis (PCA)

**Fig. 9:**
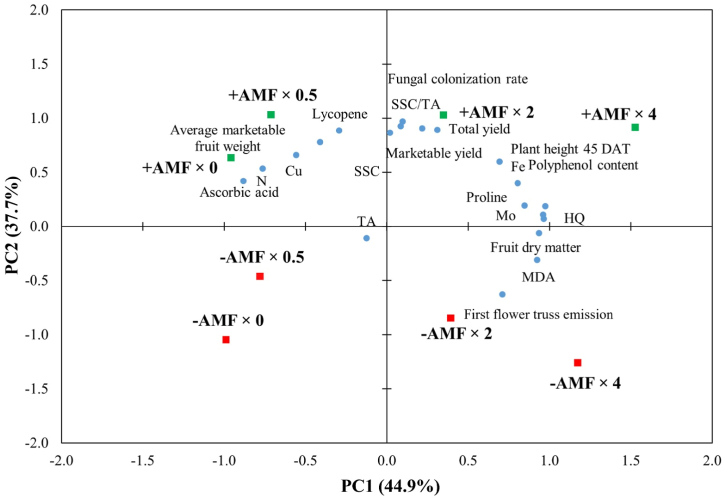
Principal component analysis (PCA) loading plots for ‘Tyty’ cherry tomato yield and qualitative traits as influenced by arbuscular mycorrhizal fungi inoculation (-AMF, +AMF) and Mo enrichment at different doses (0, 0.5, 2 and 4 μmol L^−1^). N: nitrogen; Cu: copper; Mo: molybdenum; SSC: soluble solids content; TA: titratable acidity; MDA: malondialdehyde; HQ: hazard quotient.

The principal component analysis (PCA) showed that the first two components explained 82.6 % of the total variance (Fig. 9).

**Fig. 10:**
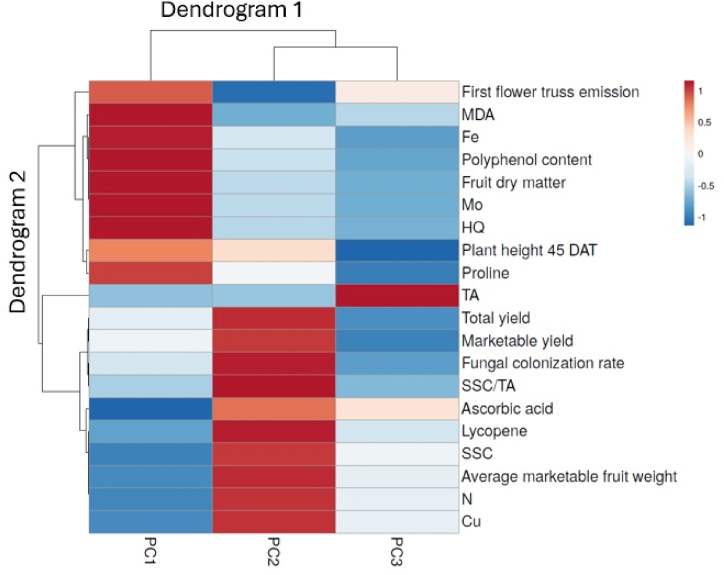
Heat map analysis including all recorded parameters in response the principal components with an eigenvalue higher than 1.0 (PC1, PC2 and PC3). The heat map figure was created using the https://biit.cs.ut.ee/clustvis/online program (accessed on March 25, 2024) package. N: nitrogen; Cu: copper; Mo: molybdenum; SSC: soluble solids content; TA: titratable acidity; MDA: malondialdehyde; HQ: hazard quotient.

The first principal component (PC1) and the second principal component (PC2) described 44.9 % and 37.7 % of the total variance, respectively. The first principal component was mainly positively related to fruit dry matter, polyphenol content, MDA, proline, Mo, Fe and HQ, while it was mainly negatively correlated to ascorbic acid and N (Supplementary Table 6). The second principal component was principally positively correlated to fungal colonization rate, total yield, marketable yield, average marketable fruit weight, SSC, SSC/TA and lycopene (Supplementary Table 6). A heat-map analysis of all tested traits was performed to summarize the influence of the principal components with an eigenvalue higher than 1 (Fig. 10).

The graphic analysis consists of two dendrograms. The first (Dendrogram 1) located at the top contains the 3 PCs, the second (Dendrogram 2) located on the left side includes all the variables that affect this distribution. Dendrogram 1 showed two main clusters, the first on the left includes PC1, the second on the right includes PC2 and PC3. In particular, the first group showed higher value of first flower truss emission, MDA, Fe, polyphenol content, fruit dry matter, Mo, HQ, plant height 45 DAT and proline than the second group. Regarding the second cluster, PC2 showed the lower Fe, polyphenol content, fruit dry matter, Mo, HQ, proline, TA, but higher total yield, marketable yield, fungal colonization rate, SSC/TA, ascorbic acid, lycopene, SSC, average marketable fruit weight, N and Cu than PC3.

The PCA loading plot was clearly divided into four quadrants (Fig. 9); all the +AMF treatments were placed in the upper side of the plot, whereas all the combinations enclosing the non-inoculated treatments were allocated in the bottom side (Fig. 9). Moreover, the PCA highlighted that Mo doses were clearly separated in two different parts of the plot; the doses 0.0 and 0.5 μmol L^−1^ were sited in the left part, while the doses 2.0 and 4.0 μmol L^−1^ were placed in the right part of the plot (Fig. 9). The combinations AMF × 0 and AMF × 0.5 were localized in the upper left side of the plot with ascorbic acid, N, Cu, average marketable fruit weight and lycopene. The combinations AMF × 2.0 and AMF × 4.0 were located in the upper right side of the plot along with SSC, SSC/TA, fungal colonization rate, total yield, marketable yield, plant height 45 DAT, Fe, Mo, polyphenol content, proline and HQ. The combinations -AMF × 0.0 and -AMF × 0.5 were allocated in the bottom left quadrant of the plot with TA. Finally, in the bottom right quadrant of the plot, the -AMF × 2.0 and -AMF × 4.0 combinations were found along with fruit dry matter, MDA and first truss emission.

## 4 Discussion

Biofortification of vegetables with trace elements makes soilless systems particularly suitable and attractive for premium quality vegetables production. However, depending on dosage and application system, trace elements can be toxic for plants. In this respect, the appropriate management of the nutrient solution is a crucial aspect of horticultural systems [62], especially in soilless cultures. At the same time, sustainable and emerging agronomic techniques, such as the employment of biostimulants, could be helpful in increasing plant tolerance to trace elements detrimental effect [63], as in the case of Mo overdosage [64]. In the current article, we investigated the interactive effects between different Mo dosages in the nutrient solutions and the arbuscular mycorrhizal fungi inoculation on plant performance, fruit quality, proline, lipid peroxidation (malondialdehyde) and Mo-biofortification safety of a cherry tomato genotype grown in a soilless system. Results showed that fungal colonization rate at the end of the experiment was significantly enhanced by the +AMF treatment. However, the highest values were observed in +AMF plants exposed to the highest Mo dosage (4.0 μmol Mo L^−1^). In this respect, Shi et al. [51] reported that Mo is an essential trace element for plants and microbes, including rhizobia and AMF. Campo et al. [65] also reported that in a soybean field crop an adequate Mo dosage increases rhizobia cells survival. Similarly, we observed an eliciting effect of Mo on fungal colonization rate, probably due to an improved arbuscular mycorrhizal fungi nutritional status. Nonetheless, the threshold values for Mo toxicity in arbuscular mycorrhizal fungi are still unexplored.

Our results are in agreement with those of De Pascale et al. [66] and Rouphael et al. [39], who claimed that the positive effects on plant growth provoked by the AMF inoculation may be related to an increased plant mineral uptake, translocation efficiency and to an enhanced root system expansion. This positive influence may be attributed to the role of Mo in plant nitrogen metabolism, which is directly associated to plant growth [28]. Remarkably, when plants were inoculated with AMF and - simultaneously - fertigated with 4.0 μmol Mo L^−1^, they showed the highest values in terms of plants height 45 DAT, whereas non-inoculated plants showed a decreasing trend when a dose of Mo higher than 2 μmol L^−1^ was supplied. This behaviour gives insight into the role of mycorrhizae in alleviating the toxic effects of high Mo doses.

Findings on first flower truss emission time are similar to those reported by Ortas et al. [67] on tomato plants. As it was revealed in this research and previously showed by Ortas [68], the AMF inoculation can increase plant adaptation to various growing conditions, such as open field or protected environment, enhancing plant growth and development. Consequently, the reduction of the first flower truss emission time could be related to a more efficient seedling behaviour during the establishment phase when plantlets grown in the nursery have to cope with the new the cultivation environment. Since plants fertigated with the highest Mo doses (2.0 or 4.0 μmol L^−1^) revealed a higher height 45 DAT than those fertigated with 0.0 or 0.5 μmol L^−1^, we may hypothesize that the flowering stage was delayed due to a higher plant vigour. These findings could have an impact on crop earliness and, consequently, on market fruit availability.

The findings on total yield and marketable yield are coherent with Balliu et al. [69], who found that the upgraded nutrition status of AMF tomato inoculated plants is due to the elicited plant nutrient uptake activity via mycorrhizal hyphae or through root system allometry alterations. Furthermore, the results are totally in agreement with those of Sabatino et al. [19], who revealed that ‘Tyty’ cherry tomato plants do not react to different Mo dosages. However, the same authors also found that the tomato response to different Mo dosages is a genotype related trait.

The positive effects of Mo on dry matter percentage could be linked to its role in N metabolism. Indeed, Conversa et al. [70] and Ronga et al. [71] reported that N supply significantly up-surge dry matter percentage of many crops such as, wheat, broccoli and tomato.

As it was revealed in this research and previously reported by Sabatino et al. [72], soluble solids content was positively influenced by the +AMF treatment. These results could be linked to AMF effect on plant primary metabolism [39] and, in general, to the improved plant nutritional status. Moreover, as reported by Cordeiro et al. [73], mycorrhized plants could have higher photosynthetic rate, as shown by the yield increase, which influenced the biosynthesis of sugars. The effect of Mo on SSC could be explained by its role in the biosynthesis of sucrose phosphate synthase and other sucrose metabolizing enzymes which have important implications in establishing the soluble sugars content in fruits of different species [74]. This affects sensory perception with possible positive impact on consumer's choice.

Findings on TA are partially in agreement with those obtained by Cordeiro et al. [73], on strawberry fruits, but disagree with those of Li et al. [74]. These conflicting results could be explained by a species related response to AMF inoculation and Mo supply.

The SSC/TA ratio is an important marker for ripe fruit sweetness. Data on SSC/TA ratio, as stated by Cordeiro et al. [73], could be related to the assumption that AMF significantly enhance SSC, while have no effect on TA. Furthermore, the results are partially in line with those of Li et al. [74], who found an increase of TSS/TA ratio when plants are treated with the highest Mo dose. However, as reported by the same authors, medium and low Mo dosages do not significantly influence SSC/TA ratio, thus, we can assume that in our experiment Mo dosages were not high enough to influence this parameter.

Outcomes on ascorbic acid are in accordance with other reports [[75], [76], [77]]. The effect of AMF on ascorbic acid could be related to the higher mineral content of mycorrhized plants, which in turn results in an increase in plant secondary metabolites [39]. Moreover, the reduction in ascorbic acid could be related to the detrimental effect of Mo at high dosages. Indeed, at 0.5 μmol L^−1^ Mo did not decrease ascorbic acid concentration in fruits, however, when Mo was supplied with a dosage over than 0.5 μmol L^−1^ a significant reduction of this parameter was recorded, highlighting that Mo supply promotes hormesis in ascorbic acid. Interestingly, our study also pointed out that plants treated with Mo and inoculated with the microbial biostimulant showed a lower reduction in ascorbic acid content. Thus, in this regard, we might speculate that AMF inoculation had a buffer effect on the high dosage of Mo.

Data on fruit polyphenols concentration agree with those of Toussaint et al. [78], who hypothesized a possible mechanism by which AMF may enhance polyphenols through the increase of N assimilation. Indeed, this improvement influences the biosynthesis of imperative amino-acids, precursors of various enzymes involved in the biosynthesis of phenolic compounds. Toussaint et al. [78] also reported the ability of AMF to modulate plant phytohormones levels, such as cytokinins and gibberellins, which are involved in polyphenols biosynthesis. We found that Mo, at a dosage higher than 0.5 μmol L^−1^, enhanced polyphenols content. Since, as reported by Šamec et al. [79], plants stress conditions trigger polyphenols biosynthesis, we may assume that in our study the polyphenols increase was linked to the toxicity caused by the high dosage of Mo supply (2.0 and 4.0 μmol L^−1^).

Results on fruit lycopene concentration are in line with those of Aguilera et al. [80] who, investigating the effect of AMF from acidic soils on tomatoes lycopene concentration, found that the lycopene increase is due to the activation of genes comprised in the biosynthesis of terpenoids. Furthermore, data showed that Mo application at high dosages significantly reduced fruit lycopene concentration. This effect could be ascribed to the Mo toxic effect. Fascinatingly, the AMF treatment decreased the negative effect of high Mo dosages. As highlighted by Flores et al. [81], this effect could be related to an enhanced Mo accumulation and, consequently, to an improved N utilization, which influences lycopene fruit concentration. Thus, the combined supply of AMF and Mo could be useful to increase the human bioactive compounds intake, such as lycopene.

The N increase in response to AMF inoculation in cherry tomato fruits is related to the development of extra-radical mycorrhizal hyphae, which enhance plant N uptake [82]. In our research, fruit N concentration decreased linearly as Mo dosages increased. The influence of Mo on N metabolism is very well known, as Mo is comprised in enzymes involved in N metabolism (assimilation and reduction) [83]. AMF inoculation and Mo biofortification significantly interacted in modulating fruit N concentration. In particular, it emerged that, independently of the Mo dosages, inoculated plants had higher N content than those non-inoculated.

Data on Fe fruit concentration could be explained by the positive effect exerted by the microbial biostimulant on plant mineral uptake [39]. In our research Mo application differently modulated Fe concentration in fruits. In particular, our data pointed out that Fe fruit concentration was enhanced when non-inoculated plants were treated with a dose up to 2.0 μmol Mo L^−1^. Whereas, in inoculated plants, Fe concentration increased even at high Mo doses. The inhibiting high dose Mo effect on Fe accumulation can be linked to the plant uptake Fe and Mo mechanisms that are very similar each other [84]. However, when plants were treated with AMF, the competition between Mo and Fe was not recorded. Indeed, the highest Fe concentration was found in inoculated plants treated with the highest Mo dose. Consequently, we might hypothesize that AMF had some important functions in the modulation of the microelement competition.

Data on Cu are partly in line with those of Tran et al. [85], who found that AMF inoculation differently modulate Cu concentration in plants. As stated by Baum et al. [86], AMF effect on plant nutrient uptake is variable among plant species and differs also by the minerals. Consequently, we can affirm that, as it was revealed in this research and previously reported by Tran et al. [85] and Darakeh et al. [87], AMF inoculation increased Cu concentration. The study also highlighted that Mo application differentially modulate Cu concentration in fruits depending on AMF inoculation. Indeed, in control plants, Mo significantly reduced fruit Cu concentration when a dose higher than 0.5 μmol L^−1^ was applied. Conversely, inoculated plants showed a different trend with a reduction in Cu concentration only in plants exposed to the highest Mo dose. Consequently, it seems that AMF inoculation mitigated the adverse effect of Mo on Cu concentration.

Findings on Mo concentration could be related to the capability of AMF to mitigate the detrimental effect of high concentration of minerals, such as Mo [51]. Indeed, control plants fertigated with the highest Mo dosage revealed lower fruit Mo concentration than those inoculated, indicating that AMF increased plant tolerance to high Mo dosages, which in turn resulted in higher Mo accumulation in fruits.

Malondialdehyde (MDA) is a marker of lipid peroxidation, which is related to plant stress [88]. Findings on MDA suggest that AMF inoculation decreased the peroxidation and, consequently reduced plant stress. Moreover, Mo doses higher or lower than 0.5 μmol L^−1^ (recommended dose) significantly enhanced MDA content. This might suggest a non-optimal Mo supply. Consequently, in both cases (higher or lower than recommended dose), we can assume that plants were under oxidative stress.

Proline is known as an environmental stress indicator for plants [89]. Results on proline are consistent with those of Liu et al. [90], who reported that AMF inoculation increase proline accumulation in plant tissues via the NO signaling molecule. Indeed, since proline is an amino acid [91], its biosynthesis is related to the availability of nitrogen, which was more available in inoculated plants. Furthermore, the Mo boosting effect on proline could be attributed to the direct role of Mo on nitrogen metabolism which influenced the proline biosynthesis. Proline content in non-biofortified plants was higher than in plants treated with 0.5 μmol L^−1^. Since proline is also considered an abiotic stress marker in several plants [92], we can hypothesize that plants cultivated without Mo are under stress condition. These outcomes are additionally corroborated by Marschner [28], who stated that Mo is an essential microelement for plant growth and development.

To assess the potential damage of Mo on human health, the hazard quotient (HQ) was calculated, as reported by USEPA protocol. Since in our experiment the HQ values were always lower than 1, we can assume that, in this experiment, the hazard was at “negligible hazard” level. Thus, a daily dose of 200 g of Mo-enriched fruits from all treatment combinations might be considered safe for human consumption.

Principal components analysis revealed a full composition for evaluating the influence of AMF inoculation and Mo supply on tomato yield, qualitative traits, lipid peroxidation, proline and hazard quotient. The treatment combinations were evidently parted via the PCA. The analysis established that several variables were influenced by the +AMF treatment in interaction with Mo doses, since these combinations were grouped in the upper side of the loading plot with almost all the variables, pointing out that AMF treatment effect on the dependent variables outclassed the Mo-biofortification influences.

**Fig. 11:**
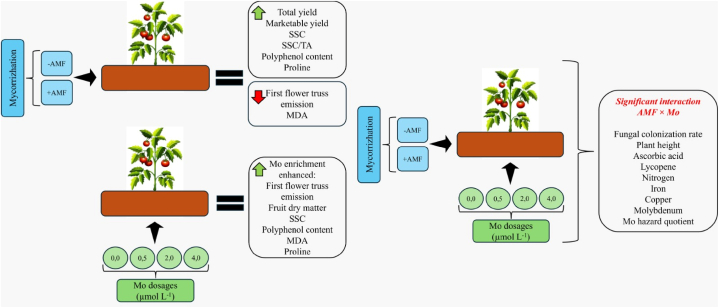
Comprehensive synthesis of the main findings of the study.

The main findings of the study were reported in Fig. 11.

## 5 Conclusions

This study indicated that Mo-biofortification combined with AMF inoculation may represent a viable agronomic protocol to enhance yield, produce premium quality tomato fruits and, concomitantly, improve Mo dose in human diet. In particular, the combination of AMF inoculation and Mo biofortification at 2.0 or 4.0 μmol L^−1^ improved fungal colonization rate, plant height, ascorbic acid, lycopene and fruit Fe, Cu and Mo concentration. The current research also showed that high Mo dosages (4.0 μmol L^−1^) might have detrimental effects on tomato plants. However, our study evidenced that AMF inoculation can mitigate this negative effect. In the light of our findings, further studies on the interaction between AMF and microelements in other vegetable crops are recommended.

## Data availability statement

Data will be made available on request.

## CRediT authorship contribution statement

**Lorena Vultaggio:** Writing – review & editing, Writing – original draft, Methodology, Investigation, Formal analysis, Data curation, Conceptualization. **Enrica Allevato:** Writing – review & editing, Writing – original draft, Investigation, Formal analysis, Data curation. **Leo Sabatino:** Writing – review & editing, Writing – original draft, Visualization, Validation, Supervision, Resources, Project administration, Funding acquisition, Data curation, Conceptualization. **Georgia Ntatsi:** Writing – review & editing, Writing – original draft, Methodology, Investigation. **Youssef Rouphael:** Writing – review & editing, Writing – original draft, Validation. **Livio Torta:** Investigation, Formal analysis. **Salvatore La Bella:** Writing – review & editing, Supervision. **Beppe Benedetto Consentino:** Writing – review & editing, Writing – original draft, Methodology, Investigation, Formal analysis, Data curation, Conceptualization.

## Declaration of competing interest

The authors declare that they have no known competing financial interests or personal relationships that could have appeared to influence the work reported in this paper.
