# Supplementary material for: Modulation of cherry tomato performances in response to molybdenum biofortification and arbuscular mycorrhizal fungi in a soilless system
Source: Heliyon. 2024 Jun 22;10(13):e33498. doi: 10.1016/j.heliyon.2024.e33498 (PMC11255863; doi:10.1016/j.heliyon.2024.e33498)
Supplement: Multimedia component 1 [file mmc1.docx]

**Table S1.** First truss emission, total yield, marketable yield and average marketable fruit weight as influenced by arbuscular mycorrhizal fungi inoculation (AMF) and Mo biofortification.

| **Treatments** | **First flower truss emission (DAT)** | | **Total yield (kg plant^-1^)** | | **Marketable yield (kg plant^-1^)** | | **Average marketable fruit weight (g fruit^-1^)** | |
| --- | --- | --- | --- | --- | --- | --- | --- | --- |
| *Mychorrhization* |  |  |  |  |  |  |  |  |
| -AMF | 25.4 | a | 1.3 | b | 1.3 | b | 30.4 | a |
| +AMF | 23.5 | b | 2.0 | a | 1.8 | a | 30.2 | a |
|  |  |  |  |  |  |  |  |  |
| *Mo doses (µmol L^-1^)* |  |  |  |  |  |  |  |  |
| 0.0 | 23.2 | b | 1.7 | a | 1.5 | a | 30.3 | a |
| 0.5 | 23.2 | b | 1.8 | a | 1.6 | a | 30.8 | a |
| 2.0 | 25.7 | a | 1.5 | a | 1.5 | a | 30.1 | a |
| 4.0 | 25.7 | a | 1.7 | a | 1.6 | a | 30.1 | a |
|  |  |  |  |  |  |  |  |  |
| Significance |  |  |  |  |  |  |  |  |
| AMF | *** | | *** | | *** | | NS | |
| Mo | *** | | NS | | NS | | NS | |
| AMF × Mo | NS | | NS | | NS | | NS | |

Means with different letters are statistically dissimilar according to Tukey HSD test at *p* ≤ 0.05. ***: significant at *p* ≤ 0.001; NS: not significant. -AMF: non-inoculated; +AMF: inoculated with arbuscular mycorrhizal fungi.

**Table S2.** Fruit dry matter, SSC, TA, SSC/TA and polyphenol content, as influenced by arbuscular mycorrhizal fungi inoculation (AMF) and Mo biofortification.

| **Treatments** | **Fruit dry matter (%)** | | **SSC (°Brix)** | | **TA (% citric acid)** | | **SSC/TA** | | **Polyphenol content (mg gallic acid eq. 100 g^-1^ dw)** | |
| --- | --- | --- | --- | --- | --- | --- | --- | --- | --- | --- |
| *Mychorrhization* |  |  |  |  |  |  |  |  |  |  |
| -AMF | 6.61 | a | 8.07 | b | 0.55 | a | 14.6 | b | 17.2 | b |
| +AMF | 6.65 | a | 8.95 | a | 0.56 | a | 16.0 | a | 17.6 | a |
|  |  |  |  |  |  |  |  |  |  |  |
| *Mo doses (µmol L^-1^)* |  |  |  |  |  |  |  |  |  |  |
| 0.0 | 6.37 | c | 7.93 | c | 0.56 | a | 15.0 | a | 16.6 | c |
| 0.5 | 6.43 | c | 9.05 | a | 0.57 | a | 15.3 | a | 16.7 | c |
| 2.0 | 6.63 | b | 8.53 | b | 0.55 | a | 15.6 | a | 17.8 | b |
| 4.0 | 7.08 | a | 8.52 | b | 0.56 | a | 15.4 | a | 18.5 | a |
|  |  |  |  |  |  |  |  |  |  |  |
| Significance |  |  |  |  |  |  |  |  |  |  |
| AMF | NS | | *** | | NS | | *** | | *** | |
| Mo | *** | | *** | | NS | | NS | | *** | |
| AMF × Mo | NS | | NS | | NS | | NS | | NS | |

Means with different letters are statistically dissimilar according to Tukey HSD test at *p* ≤ 0.05. ***: significant at *p* ≤ 0.001; NS: not significant. -AMF: non-inoculated; +AMF: inoculated with arbuscular mycorrhizal fungi.

**Table S3.** Malondialdehyde (MDA) and proline as influenced by arbuscular mycorrhizal fungi inoculation (AMF) and Mo biofortification.

| **Treatments** | **MDA (µmol g^-1^ fw)** | | **Proline (µmol g^-1^ fw)** | |
| --- | --- | --- | --- | --- |
| *Mychorrhization* |  |  |  |  |
| -AMF | 4.95 | a | 6.43 | b |
| +AMF | 4.63 | b | 6.86 | a |
|  |  |  |  |  |
| *Mo doses (µmol L^-1^)* |  |  |  |  |
| 0.0 | 4.33 | c | 6.45 | c |
| 0.5 | 4.02 | d | 5.70 | d |
| 2.0 | 5.02 | b | 6.92 | b |
| 4.0 | 5.80 | a | 7.50 | a |
|  |  |  |  |  |
| Significance |  |  |  |  |
| AMF | *** | | *** | |
| Mo | *** | | *** | |
| AMF × Mo | NS | | NS | |

Means with different letters are statistically dissimilar according to Tukey HSD test at *p* ≤ 0.05. ***: significant at *p* ≤ 0.001; NS: not significant. -AMF: non-inoculated; +AMF: inoculated with arbuscular mycorrhizal fungi.

**Table S4.** Fungal colonization rate, plant height 45 DAT, ascorbic acid and lycopene as influenced by arbuscular mycorrhizal fungi inoculation (AMF) and Mo biofortification.

| **Treatments** | | **Fungal colonization rate (%)** | | **Plant height 45 DAT (cm)** | | **Ascorbic acid (mg 100 g^-1^ dw)** | | **Lycopene (mg 100 g^-1^ dw)** | |
| --- | --- | --- | --- | --- | --- | --- | --- | --- | --- |
| Mycorrhization (M) | *Mo doses* |  |  |  |  |  |  |  |  |
| -AMF | 0.0 | 1.3 | d | 69.7 | f | 1748.7 | b | 893.7 | c |
|  | 0.5 | 1.0 | d | 73.7 | e | 1744.0 | b | 889.7 | c |
|  | 2.0 | 1.3 | d | 81.3 | b | 1657.3 | d | 889.7 | c |
|  | 4.0 | 1.1 | d | 76.3 | d | 1556.7 | f | 855.7 | d |
| +AMF | 0.0 | 76.8 | c | 74.7 | e | 1877.3 | a | 912.4 | ab |
|  | 0.5 | 75.8 | c | 77.7 | c | 1883.0 | a | 915.5 | ab |
|  | 2.0 | 80.7 | b | 87.3 | a | 1720.5 | c | 917.4 | a |
|  | 4.0 | 84.2 | a | 87.0 | a | 1583.9 | e | 909.7 | b |
|  |  |  |  |  |  |  |  |  |  |
| *Significance* | |  |  |  |  |  |  |  |  |
| AMF | | *** | | *** | | *** | | *** | |
| Mo | | *** | | *** | | *** | | *** | |
| AMF × Mo | | *** | | ** | | *** | | *** | |

Means with different letters are statistically dissimilar according to Tukey HSD test at *p* ≤ 0.05. ***: significant at *p* ≤ 0.001; **: significant at *p* ≤ 0.01. -AMF: non-inoculated; +AMF: inoculated with arbuscular mycorrhizal fungi.

**Table S5.** Nitrogen (N), molybdenum (Mo), iron (Fe), copper (Cu) and hazard quotient (HQ) as influenced by arbuscular mycorrhizal fungi inoculation (AMF) and Mo biofortification.

| **Treatments** | | **N (g 100 g^-1^ dw)** | | **Mo (mg kg^-1^ dw)** | | **Fe (mg kg^-1^ dw)** | | **Cu (mg kg^-1^ dw)** | | **HQ (EDI/RfD)** | |
| --- | --- | --- | --- | --- | --- | --- | --- | --- | --- | --- | --- |
| Mycorrhization | *Mo doses* |  |  |  |  |  |  |  |  |  |  |
| -AMF | 0.0 | 1.60 | b | 0.024 | e | 11.3 | h | 3.4 | c | 0.0005 | g |
|  | 0.5 | 1.15 | d | 0.032 | d | 15.0 | f | 3.3 | c | 0.0007 | f |
|  | 2.0 | 0.89 | f | 0.039 | c | 24.7 | c | 2.6 | e | 0.0009 | d |
|  | 4.0 | 0.71 | g | 0.063 | b | 19.0 | d | 2.4 | f | 0.0015 | b |
| +AMF | 0.0 | 1.72 | a | 0.025 | e | 12.7 | g | 3.6 | b | 0.0005 | g |
|  | 0.5 | 1.57 | b | 0.035 | d | 17.3 | e | 3.7 | b | 0.0007 | e |
|  | 2.0 | 1.40 | c | 0.044 | c | 28.4 | b | 4.3 | a | 0.0010 | c |
|  | 4.0 | 1.08 | e | 0.078 | a | 31.1 | a | 2.8 | d | 0.0018 | a |
|  |  |  |  |  |  |  |  |  |  |  |  |
| *Significance* | |  |  |  |  |  |  |  |  |  |  |
| AMF | | *** | | *** | | *** | | *** | | *** | |
| Mo | | *** | | *** | | *** | | *** | | *** | |
| AMF × Mo | | *** | | *** | | *** | | *** | | *** | |

Means with different letters are statistically dissimilar according to Tukey HSD test at *p* ≤ 0.05. ***: significant at *p* ≤ 0.001. -AMF: non-inoculated; +AMF: inoculated with arbuscular mycorrhizal fungi.

**Table S6.** Eigenvalues, variance and cumulative percentages of total variance of the three principal components (PCs) for all 20 variables.

| **Variables** | **PC1** | **PC2** | **PC3** |
| --- | --- | --- | --- |
| Plant height 45 DAT | 0.694 | 0.596 | 0.248 |
| First flower truss emission | 0.710 | -0.629 | 0.195 |
| Fungal colonization rate | 0.097 | 0.967 | -0.177 |
| Total yield | 0.219 | 0.906 | -0.166 |
| Marketable yield | 0.310 | 0.891 | -0.135 |
| Average marketable fruit weight | -0.410 | 0.777 | 0.040 |
| Fruit dry matter | 0.936 | -0.063 | -0.250 |
| SSC | 0.021 | 0.863 | 0.404 |
| TA | -0.125 | -0.111 | 0.928 |
| SSC/TA | 0.085 | 0.925 | 0.010 |
| Ascorbic acid | -0.881 | 0.418 | 0.039 |
| Polyphenol content | 0.975 | 0.186 | -0.012 |
| Lycopene | -0.292 | 0.884 | -0.017 |
| MDA | 0.925 | -0.310 | -0.143 |
| Proline | 0.847 | 0.192 | -0.395 |
| N | -0.762 | 0.534 | -0.247 |
| Mo | 0.961 | 0.107 | -0.033 |
| Fe | 0.806 | 0.396 | 0.252 |
| Cu | -0.556 | 0.658 | -0.080 |
| HQ | 0.966 | 0.068 | -0.060 |
|  |  |  |  |
| *Eigenvalue* | 8.986 | 7.537 | 1.556 |
| *Variance %* | 44.930 | 37.684 | 7.780 |
| *Cumulative %* | 44.930 | 82.614 | 90.462 |
